# Nursing in Diseases of the Throat, Nose, and Ear

**Published:** 1900-03

**Authors:** 


**Nursing in Diseases of the Throat, Nose, and Ear.** By MACLEOD YEARSLEY, F.R.C.S. Pp. x., 113. London: The Scientific Press, Limited. 1899.

The appearance in book form of this excellent series of articles by the author in "*The Hospital Nursing Mirror*" will be welcomed by those nurses whose work is largely connected with these special classes of affections, especially as they have been amplified by chapters on the elementary anatomy and physiology of the regions. The text is clear and full; the illustrations numerous and useful. The small volume contains everything that the nurse need know, and much else besides which will be found most useful by practitioners who are occasionally called upon to treat special affections of the nose, throat or ear.

